# Effect of Seaweed Fucoidan Incorporated Into Batter on Shelf Life Extension of Silver Carp (*Hypophthalmichthys molitrix*) Nugget During Refrigerated Storage

**DOI:** 10.1002/fsn3.72070

**Published:** 2026-06-30

**Authors:** Sedigheh Babaei, Farzaneh Vardizadeh, Mahshid Mojarrad, Tahereh RoshanZamir, Samaneh Rajabloo, Mehrnaz Ghanbarzadeh

**Affiliations:** ^1^ Department of Natural Resources and Environmental Engineering, School of Agriculture Shiraz University Shiraz Iran; ^2^ Fisheries Department, Faculty of Natural Resources University of Guilan Sowmeh Sara Guilan Iran; ^3^ Department of Food Science and Technology, School of Agriculture Shiraz University Shiraz Iran; ^4^ Department of Food Hygiene and Public Health, School of Veterinary Medicine Shiraz University Shiraz Iran

**Keywords:** fucoidan seaweed, refrigerated storage, sensory evaluation, shelf life, silver carp nugget, sulfated polysaccharide

## Abstract

This study aimed to use fucoidan from *Sargassum angustifolium* directly in batter (1%, 2%, and 3% w/w; F1, F2, and F3, respectively) of silver carp ( *Hypophthalmichthys molitrix* ) nuggets, to investigate their properties during the storage (4, 8, 12, and 18 days) at 4°C. The results of chemical analysis showed no significant difference in moisture and ash content of the different treatments (*p* > 0.05). The TVB‐N content increased until day 12 and then decreased on day 18 (*p* < 0.05), remaining generally within acceptable limits. TBA content also increased during the period, but no significant differences were observed among the treatments at the end of the period (*p* > 0.05). Fucoidan significantly reduced the microbial loads of Enterobacteriaceae, psychrophiles, molds, and yeasts in fish nuggets, and as fucoidan concentration in the nugget batter increased, its inhibitory effect also improved (*p* < 0.05). The highest values of total color changes (ΔE) and lightness (L*), red‐green (a*), and yellow‐blue (b*), were observed in treatments containing fucoidan, especially in nuggets containing 3% fucoidan (F3). Sensory evaluation also showed that the F3 was the best in terms of color, odor, texture, taste, and overall acceptance. Overall, the incorporation of fucoidan into nugget batter (especially at 3%) can improve microbial stability and maintenance of quality attributes, suggesting its potential as a functional ingredient for extending the refrigerated shelf life of fish‐based products. So, it could have potential applications in the food industry.

## Graphical abstract

The fucoidan from *Sargassum angustifolium* incorporated directly in batter (0%, 1%, 2%, and 3% w/w; C, F1, F2, and F3, respectively) of silver carp ( *Hypophthalmichthys molitrix* ) nuggets increased some physiochemical properties during the refrigerated storage.

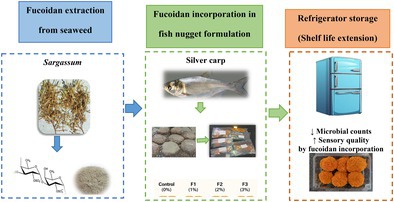

## 1 Introduction

The increase in global population and the industrialization of societies have increased the desire to consume ready‐to‐eat products. Among ready‐to‐eat meat products, marine products such as fish have a special place due to their nutritional value, including high content of essential n‐3 PUFA and essential amino acid content (Uçak et al. 2011). So, the food industry is trying to produce new types of products with new formulas, such as fish burgers, finger foods, fish nuggets, fish sausages, etc., from bycatch and low‐consumption species, while improving their desirable sensory properties (Ali et al. 2019). Many studies have been conducted on the preparation and evaluation of texture and sensory properties of various products, including minced fish ( *Pangasius hypophthalmus* ) (Mahato et al. 2024), tilapia burger ( *Oreochromis niloticus* ) (Bainy et al. 2015), and grass carp burger ( *Ctenopharyngodon Idella* ) (Haq et al. 2013).

Fish nuggets are a meaty product produced by shaping boneless minced fish, which are subjected to flouring, glazing, and breadcrumb coating, respectively, and then pre‐fried in oil and packaged, frozen, and stored. However, fish and their products can undergo adverse changes during storage, which can reduce the shelf life of the products. Spoilage mechanisms such as microbial growth, lipid oxidation, internal enzyme activity, nonenzymatic browning, and enzymatic browning cause these changes (Medina et al. 2009). For example, during the progressive oxidation reaction, unsaturated fatty acids react with oxygen through a free radical chain mechanism and form volatile compounds such as hydroperoxides, fatty acids, ketones, etc., which change the organoleptic properties of the food and reduce their nutritional value as well as shelf life (Feng et al. 2021). Therefore, in seafood products, the use of antimicrobial and antioxidant agents is often essential to increase their shelf life and also to achieve other beneficial effects.

Recent research on algae as natural antioxidant and antibacterial agents has mainly focused on the targeted extraction of natural and bioactive compounds, one of which is sulfated polysaccharides extracted from marine macroalgae cell walls (Deepika and Nadu 2017). One of the types of sulfated polysaccharides extracted from Sargassum, a brown macroalgae (Phaeophyceae), is Fucoidan (Vo and Kim 2013), generally extracted with hot water and alcohols, and has antioxidant capacities as well as specific antimicrobial effects (Roohinejad et al. 2017). Due to the hard plant cell wall, the extraction steps must be properly performed. Fucoidan is a nontoxic component containing sulfate and various compounds, including galactose, xylose, fucose, and glucuronic acid (Elizondo‐Gonzalez et al. 2012), which has some properties such as suspension, film and gel formation, and emulsion stabilization. Therefore, it has the potential to be used in the formulation of functional foods and food/nutraceutical supplements (Rioux et al. 2009).

Previous studies have shown that the seaweed dried powder and its extracts improve the quality and shelf life of seafood products (Mamat et al. 2024; Goswami et al. 2025; Patel et al. 2025). Asadi Farsani et al. (2018) reported the effect of brown algae extract *Iyengaria stellate* on the shelf life of rainbow trout ( *Oncorhynchus mykiss* ) nugget fillets. Jannat‐Alipour et al. (2018, 2019) studied the effects of sulfated polysaccharides of green algae *Ulva intestinalis* on physicochemical properties of silver carp ( *Hypophthalmichthys molitrix* ) surimi, and the effects of adding the algae powder and its sulfated polysaccharide on increasing the shelf life of fish surimi, respectively. Moreover, Albertos et al. (2019) reported the half‐life extension of rainbow trout burgers coated with chitosan edible film prepared with *Himanthalia elongata* and *Palmaria palmata* seaweed extracts. Similar results were reported by Hentati et al. (2019) about *Barbus barbus* fish burgers enriched with 1% *Jania adhaerens* and *Champia compressa* algae supplements.

A few studies have been conducted on the use of fucoidan sulfated polysaccharide to increase the shelf life of fishery products, including the effects of fucoidan on the physicochemical and antioxidant properties of surimi products (Zheng et al. 2021), the effects of layer by layer (LbL) coatings (combination of fucoidan in chitosan‐alginate LbL coatings) on increasing the shelf life of rainbow trout fillets (Khorami et al. 2024), and the effects of edible coatings of chitosan and fucoidan with hydrolyzed carp protein on increasing the shelf life of sturgeon fillets (Malekkolaei et al. 2025).

Given the limited studies, this study aimed to use fucoidan extracted from the brown algae *Sargassum angustifolium*, a native alga of the Persian Gulf, directly in the batter of silver carp nuggets and to investigate the microbial, chemical, mechanical, and sensory attributes of the nuggets during an 18‐day storage period (Khorami et al. 2024) in the refrigerator.

## 2 Materials and Equipment

The Silver carp ( *Hypophthalmichthys molitrix* ), fish protein isolate (FPI), potatoes, wheat flour, onion, garlic, spices, rice flour, starch, breadcrumbs, fucoidan from Sargassum, methyl red, boric acid, TBA reagent, HCl, trichloroacetic acid (TCA), MgO, normal saline, Plate Count Agar (PCA), VRBD (Violet Red Bile Dextrose) agar, Sabouraud Dextrose agar (SDA), and stomacher bags were purchased from different trusted companies.

For analysis some equipments such as meat grinder (MG‐1400R, Pars Khazar, Iran), incubator (503, Arian Azma Teb, Iran), vortex (Vargha Tajhiz, Iran), microbial hood (JTLVC2X, Jhal Tajhiz, Iran), oven (KM23S, Fara Azma, Iran), spectrophotometer (T70, PG Instruments Limited, England), autoclave (RT‐2, Reyhan Teb, Iran), scale (ENTRIS224‐1S, Sartorius, Germany), micro‐Kjeldahl (Buchi, Switzerland), ELISA (Cytation 3 imaging, Viragen Cell Identification Solution, Iran), electric oven (Atbin, Iran), freeze dryer (B‐5003‐FD LTD, Dena Vacuum Industry, Iran), pH meter (PTR79, Zag Chemie Co., Iran), blender (HB‐5503AP, Iran), rotary (Fara Azma, Iran), refrigerator (JTCL, Jhal Tajhiz, Iran), freezer (Pars Khazar, Iran), millpore water distillation system (QUV3‐Direct, France) and colorimeter (MAT 2000, Iranian Teb Barez, Iran) were used.

### 2.1 Sulfated Polysaccharide Extraction (Fucoidan)

Fucoidan was extracted based on the Vardizadeh et al. (2021) method. Briefly, pigments were removed using ethanol, and the *Sargassum angustifolium* was washed several times with acetone. The decolorized seaweed was mixed with distilled water and heated at 70°C for 3–4 h and then centrifuged for 10 min at 5000 rpm. The supernatant was collected, and calcium chloride was used to remove alginate. Finally, cold ethanol (96%) was added to the supernatant and maintained in the refrigerator overnight. The precipitated fucoidan was washed with acetone and ethanol and eventually dried.

### 2.2 Treatments and Fish Nugget Preparation

Twenty‐five fresh silver carp (average weight 650 g) were caught from the fish farm, and after skinning, deboning, and filleting, they were transferred beside the ice to the fish processing laboratory.

After washing, the fish were frozen in a ziplock bag for 24 h at −18°C. Also, in this study, fucoidan was extracted from Sargassum algae according to previous studies (Vardizadeh et al. 2021), and the isolated protein was extracted from Siberian sturgeon ( *Acipenser baerii* ) waste in previous studies (Babaei et al. 2024), whose physicochemical characteristics had been proven and were used.

To prepare fish nuggets, boneless silver carp fillets (43%), FPI (25%) (total of mince fish and FPI was 68%), and boiled potatoes (19.3%) were completely ground using the meat grinder, and other spices including wheat flour (4%), grated onion (3%), grated garlic (0.5%), salt (1.3%), white pepper (0.5%), red pepper (0.4%), rosemary (0.4%), coriander (0.2%), ginger (0.1%), galangal (0.3%), turmeric (0.1%), cloves (0.1%), and baking powder (2%) were added and kneaded well. After preparing the fish nuggets, they were molded (5 cm in diameter and about 1 cm in height), and placed in a freezer at −18°C for 1 h.

Then, the nuggets were dipped in a fucoidan‐treated batter. The batter was prepared from wheat flour (66%), rice flour (15%), starch (17%), salt (2%), and water (in a ratio of 1:1.5, w: v). The treatments in this study were as follows: (the added amount of fucoidan was replaced by a decrease in the weight of wheat flour).

− Control: Fish nuggets without fucoidan in the batter
− F1: Fish nuggets prepared with a batter containing 1% fucoidan (w/w)
− F2: Fish nuggets prepared with a batter containing 2% fucoidan (w/w)
− F3: Fish nuggets prepared with a batter containing 3% fucoidan (w/w)

After glazing, the surface of the nuggets was covered with fine breadcrumbs and stored again at −18°C for 30 min. The preliminary cooking stage was performed at 180°C for 120–180 s, and then the nuggets were placed in labeled containers and stored in the refrigerator at 4°C. Sampling was performed randomly on days 0, 4, 8, 12, and 18. On the sampling days, the nuggets were removed from the freezer and thawed at room temperature, then deep‐fried at 190°C for 3 min and subjected to chemical and sensory evaluation. All the analyses were carried out in three replicates.

### 2.3 Proximate Analysis

The nugget composition (protein, moisture, and ash) of the samples was measured according to standard methods (AOAC 2000). Protein content was measured using a micro‐Kjeldahl (Buchi, Switzerland), moisture content at 105°C using an oven (KM23S, Fara Azma, Iran), and ash content by combustion in an electric oven (Atbin, Iran) at 520°C. Fat content was measured using the Hannon Soxhlet (SOX406) method according to standard 742, and by the dried sample used.

### 2.4 Chemical Analysis

#### 2.4.1 pH Value

The pH value was assessed using the pH meter (PTR79, Zag Chemie Co., Iran).

#### 2.4.2 Total Volatile Base Nitrogen (TVB‐N)

The TVB‐N content was determined following the procedure described by Jamróz et al. (2019) with a Kjeldahl flask (Buchi, Switzerland), and the results were expressed as mg of nitrogen per 100 g of nugget. The TVB‐N value was finally obtained using the following equation:

(1) $$\mathrm{TVB}-N\;\left(\mathrm{mg}\;N/100\;g\right)=14\times \mathrm{HCl}\;\left(\mathrm{mL}\right)$$

#### 2.4.3 Thiobarbituric Acid Reactive Substances (TBARS)

TBARS analysis was performed according to the procedure of Ehsani et al. (2020), with adjustments. The malondialdehyde (MDA) standards (0–5 ppm) were used to create a calibration curve. For each determination, 5 g of the sample was added to 9 mL of a stock mixture containing TBA (0.375%, w/v), TCA (15%, w/v), and HCl (0.25 M) and then heated at 90°C. The absorbance of the resulting solution was recorded at 532 nm using a spectrophotometer (T70, PG Instruments Limited, England). TBARS values were expressed as mg malondialdehyde per kg of nugget.

### 2.5 Microbial Analysis

Microbial analysis included the number of viable mesophilic aerobic bacteria, psychrophilic bacteria, Enterobacteriaceae, and molds and yeasts. For microbial analysis, 10 g of whole minced nugget was aseptically mixed with 90 mL of peptone water (0.1%) for 2 min in a blender, and serial dilutions were prepared (Basiri et al. 2015). The count of viable mesophilic aerobic bacteria and psychrophilic bacteria was performed using the PCA medium and incubated for 48 h at 37°C and 10 days at 7°C, respectively. For Enterobacteriaceae growth, the VRBD medium was used, and then incubated for 24 h at 37°C. The SDA medium was used for the count of molds and yeasts, and the plates were incubated at 25°C for 3–5 days. Logarithm of CFU/g was used to indicate the microbial count (Basiri et al. 2015).

### 2.6 Color Measurement

The color of samples was measured based on the CIELab scale and using a colorimeter (MAT 2000, Iranian Teb Barez, Iran). The samples were examined six times. Total color change (ΔE) was calculated using the following equations (Vardizadeh et al. 2021):

(2) $$\mathrm{\Delta E}={\left[{\left(L^{*}-L^{*}0\right)}^{2}+{\left(a^{*}-a^{*}0\right)}^{2}+{\left(b^{*}-b^{*}0\right)}^{2}\right]}^{0.5}$$

where L* (lightness), a* (red‐green), and b* (yellow‐blue) are the parameters of color measured on the 18th day, and L*_0_, a*_0_, and b*_0_ represent the values of the same parameters measured on day 0.

### 2.7 Sensory Evaluation

A group of 20 semi‐trained panelists familiar with seafood quality performed the sensory assessment using 5 hedonic point scales. The panelists participated voluntarily and provided informed consent before participation. The evaluation relied on subjective evaluations of color, flavor, texture, odor, and overall acceptability. Each attribute was rated on a five‐point scale. Level 1 was considered the lowest quality, and Level 5 the highest (Khorami et al. 2024). Before evaluation, all samples were assigned alphabetical codes and presented in clear containers after undergoing final frying in a deep fryer for 3 min at 190°C.

### 2.8 Statistical Analysis

Different experimental treatments were statistically compared using a one‐way ANOVA, and differences among means were further examined with Duncan's multiple range test at a 95% confidence level. The data processing was conducted using IBM SPSS Statistics (version 21).

## 3 Results and Discussion

### 3.1 Proximate Composition

The proximate analysis of processed food products, including nuggets, is an important factor that affects the quality of these products. For example, protein concentration extremely affects gel properties. Also, low lipid content products due to less chance of lipid spoilage may have a longer shelf life (Jin et al. 2007). The proximate composition of nuggets at the first (0) and last (18) days of the storage period is presented in Table 1. Based on the results, no significant difference was observed between the moisture content of treatments at the first and last days of storage (*p* > 0.05). Although at the end of the storage period, treated nuggets with fucoidan showed higher moisture content compared with the control, and the highest amount was related to F3, the differences were not significant. The results show that the use of fucoidan in batter can have a positive effect on maintaining the moisture content of samples during the storage period. Previous studies on the physicochemical properties of seaweeds have shown that polar groups of the sulphated polysaccharides, such as the carboxyl, sulfate groups, and hydroxyl, can form hydrogen bonds with water to retain moisture (Shao et al. 2015; Yang et al. 2020). The positive effect of dietary fibers on the moisture content of processed fish surimi products during a 6‐month storage period in the freezer (Jannat‐Alipour et al. 2019) and on the moisture content of frozen beef patties has been reported (Kim et al. 2016). The polysaccharide chains may form a network that has a critical role in moisture retention. Therefore, given the high‐water holding and binding capacity of dietary fibers, including fucoidan (Choi et al. 2012), meat products can improve the water and lipid binding properties by adding seaweed or its extract, which makes them ideal to be utilized in the food industries (Yaich et al. 2014).

**TABLE 1:** Proximate analysis of fish nuggets during refrigerator storage.

Test	Moisture (%)		Ash (%)		Fat (%)		Protein (%)	
Day	0	18	0	18	0	18	0	18
Control	63.7 ± 4.6	61.8 ± 3.3	5.2 ± 0.5	3.8 ± 0.1	9.0 ± 0.1^b^	7.6 ± 0.1^d^	12.2 ± 0.2^a^	14.4 ± 0.2^a^
F1	63.5 ± 0.8	62.0 ± 0.3	5.0 ± 0.7	4.2 ± 0.7	9.1 ± 0.1^b^	8.6 ± 0.1^b^	11.5 ± 0.1^b^	13.9 ± 0.0^b^
F2	64.4 ± 1.8	61.8 ± 0.4	4.6 ± 0.8	3.8 ± 0.0	8.3 ± 0.6^b^	8.1 ± 0.1^c^	11.4 ± 0.1^b^	13.7 ± 0.0^b^
F3	62.0 ± 0.7	65.4 ± 2.3	4.8 ± 0.7	3.8 ± 0.8	11.5 ± 0.1^a^	11.4 ± 0.2^a^	12.3 ± 0.1^a^	12.2 ± 0.0^c^

*Note:* Data are expressed as mean ± SD (*n* = 3). Different superscript letters in a column indicate significant differences in means between treatments (*p* < 0.05). Control (nuggets without fucoidan), F1 (nuggets containing 1% fucoidan), F2 (nuggets containing 2% fucoidan), F3 (nuggets containing 3% fucoidan).

According to Table 1, the protein contents of the control nugget and the nuggets containing 1%, 2%, and 3% of fucoidan were %14.4 ± 0.2, %13.9 ± 0.0, %13.7 ± 0.0, and %12.2 ± 0.0, respectively, at the end of the period. By adding fucoidan, the protein contents of the nuggets indicated a slight decreasing trend, which could be against the increase in moisture and lipid content of the nuggets. Based on the studies conducted, the increase in protein levels at the end of the period compared to the beginning of the period can be influenced by the amount of water lost from the ingredients (Nadia et al. 2023; Oppong et al. 2021). In the present study, given that at the end of the storage period, the numerical value of moisture, or in other words, the water holding capacity, was higher in F3 than in other treatments, the protein content in it was also lower. Studies also have shown that the protein content in fucoidan is very low (Mensah et al. 2023). Similar results were reported by da Silva et al. (2021) regarding a decrease in protein content in Nile tilapia ( *Oreochromis niloticus* ) fish nuggets coated with tapioca flour compared to the control, and by Zheng et al. (2021) in surimi products, with an increase in fucoidan percentage.

At the end of the storage period, the highest lipid content was observed in F3 (%11.4 ± 0.2) and the lowest in the control (%7.6 ± 0.1) (*p* < 0.05). Studies conducted previously have shown that the addition of fucoidan can increase emulsion stability (Chang and McClements 2015; Sun et al. 2024). Due to the function of fucoidan in the batter, it can help prevent the release of lipid from fish tissue during cooking by creating a protective layer, stabilizing the lipid emulsion, and increasing lipid content. The results of this study were in accordance with the results of Jannat‐Alipour et al. (2019) and Asadi Farsani et al. (2018) about increasing the lipid content of the treatments by adding *Ulva intestinalis* powder, and increasing the percentage of *Iyengaria stellate* extract, respectively.

The ash content of a food product indicates its mineral content. According to the results presented in Table 1, on the first and last day of the storage period, the ash contents of the treatments were between 3.4% and 8.2%, and no significant difference was observed (*p* > 0.05), indicating the limited amount of fucoidan used in the nugget batter. Similar results were reported by da Silva et al. (2021) regarding the absence of significant differences in the ash content of Nile tilapia ( *Oreochromis niloticus* ) nuggets coated with gluten‐free flours (tapioca and coconut flours) and Dehghan Tanha et al. (2021) regarding the ash content of sausages coated with sturgeon skin gelatin and extract of *Portulaca oleracea* .

### 3.2 Chemical Analysis

#### 3.2.1 pH

**FIGURE 1:**
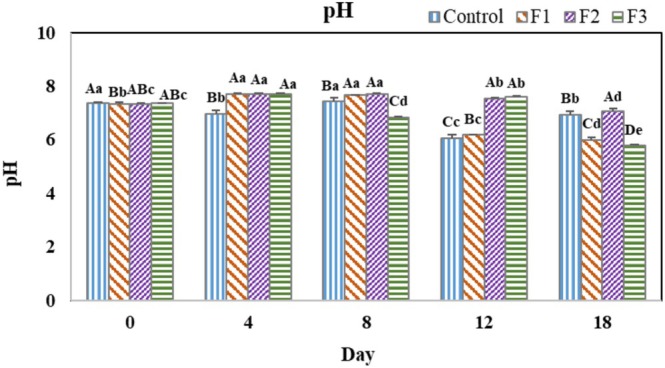
pH changes of fish nuggets during refrigerator storage. The effect of the storage period on each treatment is shown in lowercase letters. Comparisons between different treatments are shown in uppercase letters (*p* < 0.05). Data are expressed as mean ± SD. Control (nuggets without fucoidan), F1 (nuggets containing 1% fucoidan), F2 (nuggets containing 2% fucoidan), F3 (nuggets containing 3% fucoidan).

The pH changes in different groups during the storage period are shown in Figure 1. Initially, all groups had a pH value of 7.4 (*p* > 0.05), and an increasing trend was observed in the pH of the groups until day 8 (*p* < 0.05), and then in some treatments there was a decreasing trend until the end of the storage period (day 18) (*p* < 0.05). The production of volatile bases due to protein degradation by microbial and enzymatic activities led to an increase in pH (Jeyakumari et al. 2016). Jeyakumari et al. (2021) showed that the storage period had a significant effect on pH, causing an increase in the pH of *Nemipterus japonicus* nuggets treated with green tea extract and Butylated hydroxytoluene (BHT). Similar results were also observed by da Silva et al. (2021) on *Oreochromis niloticus* nuggets treated with tapioca and coconut flour during storage at −18°C. In general, although some fluctuations in the pH of the treatments were observed on different days of the storage period, at the end of the period, F3 had the lowest pH (*p* < 0.05), which can be ascribed to the antimicrobial properties of fucoidan and its ability to reduce enzymatic reaction (Arfat et al. 2015). Also, the lower pH observed in F3 compared to the control at the end of the period can be attributed to the structural features of fucoidan, which contains acidic functional groups (Wei et al. 2013). Investigation of the pH of *Barbus barbus* burgers enriched with 1% *Jania adhaerens* and *Champia compressa* showed that treatments containing algae supplements had a lower pH than the control, which was ascribed to the acidic nature of the algae (Hentati et al. 2019). Similar results were also reported by Choi et al. (2012) regarding the reduction in pH value of pork patties by adding *Laminaria japonica algae* powder, who describe such a trend to the acidic components of alginic acid and fucoidan.

#### 3.2.2 TVB‐N

**FIGURE 2:**
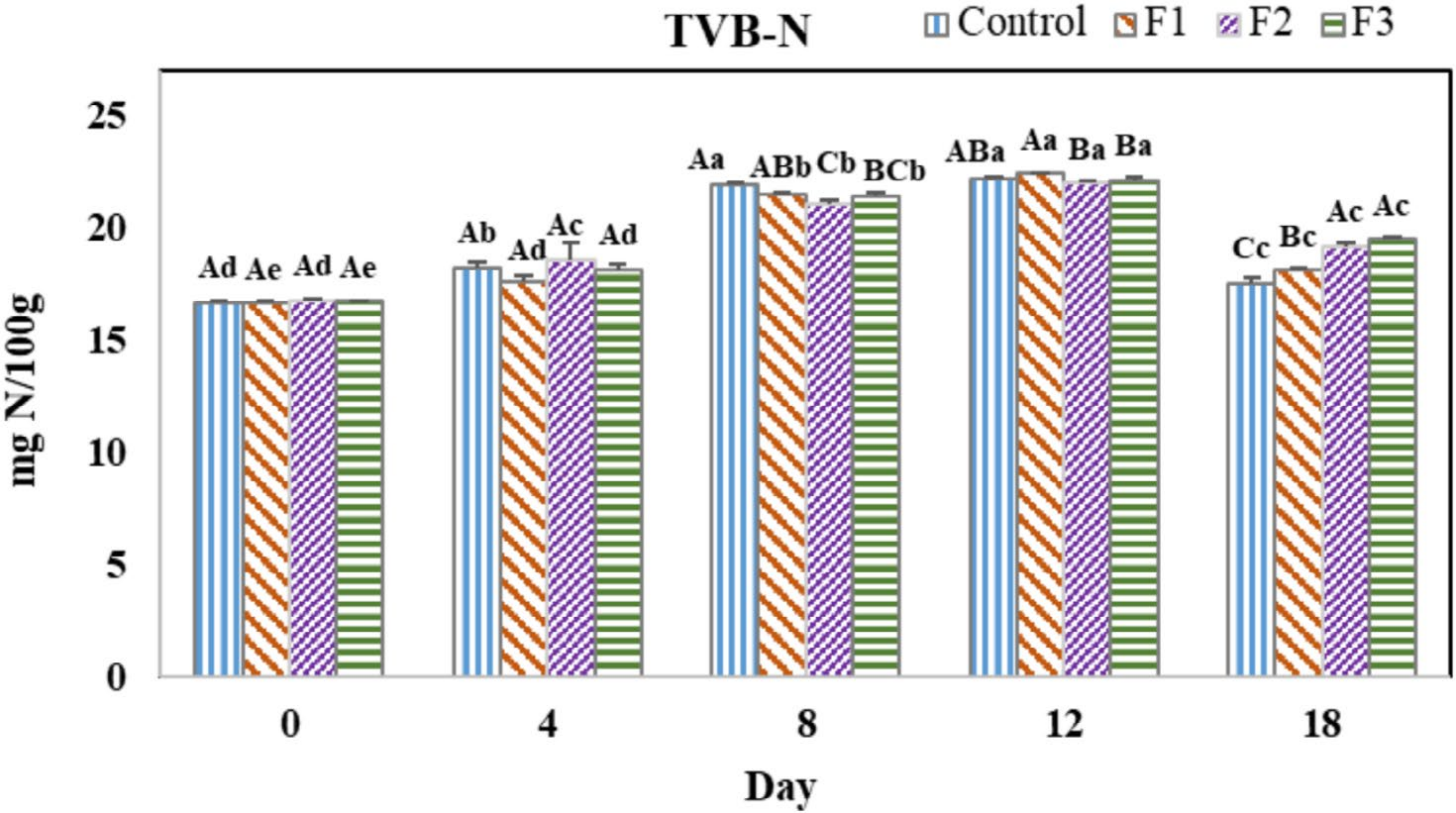
TVB‐N changes of fish nuggets during the refrigerator storage. The effect of the storage period on each treatment is shown in lowercase letters. Comparisons between different treatments are shown in uppercase letters (*p* < 0.05). Data are expressed as mean ± SD. Control (nuggets without fucoidan), F1 (nuggets containing 1% fucoidan), F2 (nuggets containing 2% fucoidan), F3 (nuggets containing 3% fucoidan).

Examining changes in TVB‐N is one of the most common indicators used to evaluate the quality of meat and meaty products, especially for fish (Weng et al. 2020). When meat products undergo microbial spoilage, their protein is broken down, and volatile base compounds such as ammonia, triethylamine, etc. are produced (Rodrigues et al. 2013). Microorganisms, autolytic enzymes, deamination of amino acids, and nucleotides play a major role in TVB‐N production (Berizi et al. 2018). Figure 2 shows the changes in TVB‐N levels in different treatments during the storage period. The highest acceptable limit for TVB‐N has been reported as 30 mg N/100 g sample (Bilgin and Gençcelep 2015), and in this study, TVB‐N was at acceptable levels in all treatments until the end of the experiment. Initially, the average TVB‐N amount of the treatments on the first day was between 6.8–16.16 mg N/100 g sample, which increased during the storage time (*p* < 0.05). The increase in TVB‐N content can be attributed to the bacteria and internal enzyme activities, which lead to the production of amine components (Berizi et al. 2018), which were also consistent with pH changes (Figure 1). Similar results indicating an increase in TVB‐N content during the storage period were observed by Khorami et al. (2024) in rainbow trout ( *Oncorhynchus mykiss* ) fillets coated with fucoidan in chitosan‐alginate LbL film during 16 days of storage at refrigerated temperature. Similar results were also reported in the study of Jeyakumari et al. (2021) on *Nemipterus japonicas* fish nuggets treated with green tea extract and BHT, Raeisi et al. (2020) on rainbow trout fillets coated with chitosan and alginate with essential oils of *Zataria multiflora*, *Mentha piperita*, and *Artemisia dracunculus* , and Sarabi et al. (2017) on fried *Lipidocybium flavobrunium* fish fillets containing rosemary extract and BHT during storage. The higher TVB‐N level and lower protein level in the fucoidan‐containing treatments compared to the control at the end of the period indicate that in these treatments, a part of the protein was degraded and converted into volatile nitrogen compounds.

#### 3.2.3 TBARS

**FIGURE 3:**
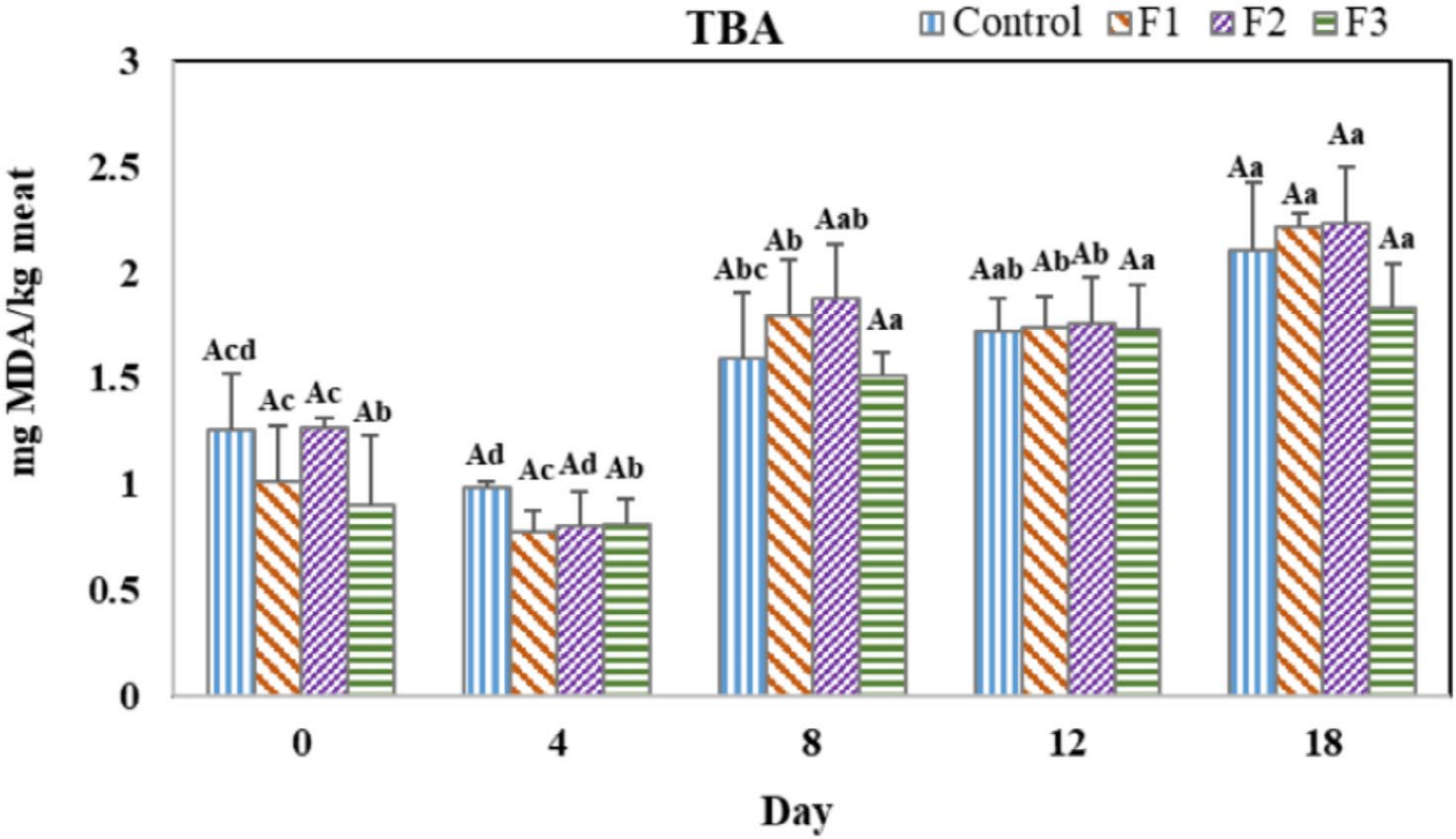
TBA changes of fish nuggets during the refrigerator storage. The effect of the storage period on each treatment is shown in lowercase letters. Comparisons between different treatments are shown in uppercase letters (*p* < 0.05). Data are expressed as mean ± SD. Control (nuggets without fucoidan), F1 (nuggets containing 1% fucoidan), F2 (nuggets containing 2% fucoidan), F3 (nuggets containing 3% fucoidan).

Fish tissue has a high susceptibility to oxidation because it contains large amounts of polyunsaturated fatty acids and hemoglobin (prooxidant) (Rico et al. 2020; Aberoumand and Baesi 2020). Lipid oxidation is a major factor for spoilage in fishery products, negatively affecting protein function and causing changes in appearance, color, odor, and off‐flavor of products (Al‐Hijazeen et al. 2016). TBARS test, also known as a fish freshness indicator, is one of the most common analyses for evaluating secondary lipid oxidation products such as malondialdehyde (MDA) (Khojah 2020). The results regarding TBARS changes in fish nuggets are shown in Figure 3. The initial TBARS value of the treatments was between 0.90–1.26 mg MDA/kg meat, and it gradually increased during the storage period (*p* < 0.05) and reached 1.83–2.23 mg MDA/kg meat at the end of the period. According to Mol et al. (2023), who reported TBARS values of 3, 5, and 8 mg MDA/kg meat as excellent, good, and acceptable limits, respectively, the TBARS values of all treatments were excellent during the storage period. at the first day, no significant difference was observed between treatments (*p* > 0.05). TBARS levels decreased in all treatments on the 4th day and ranged from 0.77 to 0.98 mg MDA/kg meat, then an increasing trend was observed in the following days until the end of the period. This increase could be due to partial dehydration of the nuggets, oxidation of unsaturated fatty acids, and volatile metabolites production due to penetration of oxygen (Chidanandaiah and Chidanandaiah Keshri 2009). The results were similar to those of Khorami et al. (2024), Delfino et al. (2023), Ditudompo et al. (2022), and Albertos et al. (2019) regarding the increase in TBARS level of products during the storage period. The results also showed that fucoidan reduced the TBARS levels of the samples compared to the control during the storage; however, these differences were not significant (*p* > 0.05). This partial decrease in lipid oxidation and TBARS levels in F3 on different days can be attributed to the high antioxidant effect of fucoidan and the coating's prevention of oxygen penetration (Pouralkhas et al. 2023; Gomaa et al. 2018). In fact, based on other studies, polysaccharides containing hydrogen donor groups (−SO_3_H and −COOH) could decrease the free radicals (Rocha de Souza et al. 2007; Vardizadeh et al. 2021). Rico et al. (2020) and Albertos et al. (2019) reported that chitosan films containing sea fennel extract and seaweed extract, respectively, reduced lipid oxidation in fish burgers, which was due to the antioxidant activity of the phenolic compounds of the extracts. Dewi and Purnamayati (2019) also stated that coating catfish sausage with different amounts of carrageenan containing garlic essential oil resulted in a reduction in TBARS and lipid oxidation during 10 days of storage. Asadi Farsani et al. (2018) showed that the use of brown seaweed (*Iyengaria stellata*) extract in rainbow trout nuggets during storage at −18°C reduced TBARS levels, which they attributed to the antioxidant effect of the seaweed extract.

### 3.3 Microbial Analysis

**FIGURE 4:**
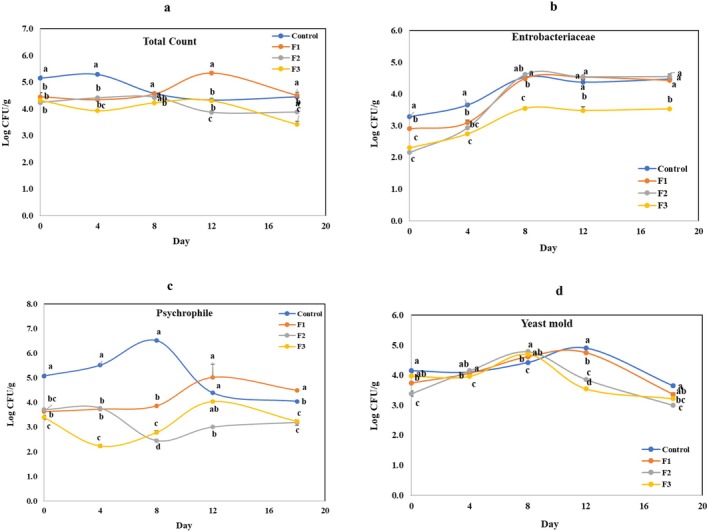
Changes in growth of total bacteria count (a), Enterobacteriaceae (b), psychrophilic bacteria (c), and yeast and mold (d) of fish nuggets during the refrigerator storage. The effect of the storage period on each treatment is shown in lowercase letters. Comparisons between different treatments are shown in uppercase letters (*p* < 0.05). Data are expressed as mean ± SD. Control (nuggets without fucoidan), F1 (nuggets containing 1% fucoidan), F2 (nuggets containing 2% fucoidan), F3 (nuggets containing 3% fucoidan).

The main cause of fish spoilage is microbial growth, and its metabolism results in the formation of amines, ketones, alcohols, and organic acids with off‐flavor components. Figure 4 shows the results of microbial growth of fish nuggets coated with fucoidan. The results showed that fucoidan had a significant effect on reducing the microbial load of fish nuggets, and with increasing fucoidan concentration in the nugget batter, its inhibitory effect also improved (*p* < 0.05). According to Figure 4a, the total bacterial count on day 0 ranged between 4.3–5.2 log CFU/g, and F2 and F3 had lower bacterial counts (*p* > 0.05). On days 4, 8, and 18 of the storage period, F3 also had a significantly lower microbial load compared to other treatments (*p* < 0.05). At the end of the experiment, F3 had a significantly lower microbial load (3.5 log CFU/g) compared to other treatments (*p* < 0.05). Nonlinear trends in microbial growth have been reported in several studies. The observed temporary decrease in microbial counts at later storage stages may be attributed to the accumulation of inhibitory metabolites and the sustained antimicrobial effect of fucoidan. Seaweeds can control bacterial growth by stopping cell division by producing secondary metabolites with antimicrobial properties, such as phenols, terpenes, and phlorotannins (Pina‐Pérez et al. 2017). Fucoidan, derived from algae cells, also has antimicrobial activity based on its amount in the batter. Fucoidan has antimicrobial effects on both Gram‐negative and Gram‐positive bacteria; however, Gram‐positive bacteria have been reported to be more sensitive to fucoidan (Pouralkhas et al. 2023; Ayrapetyan et al. 2021; Palanisamy et al. 2019). Albertos et al. (2019) showed that the use of the chitosan edible film prepared with seaweed extract significantly reduced microbial growth of rainbow trout burgers. Ehsani et al. (2020) reported that chitosan, alginate, and gelatin coatings containing lactoperoxidase and sage essential oil significantly decreased the microbial growth of common carp ( *Cyprinus carpio* ) burgers. However, physicochemical changes such as reduced water activity and oxygen limitation may contribute to fluctuations in microbial growth patterns. According to Figure 4b, the initial Enterobacteriaceae count of fish nuggets was 2–3 log CFU/g, which showed an increasing trend until day 8 of the period and then remained constant until the end of the period. The increase in Enterobacteriaceae growth in F3 was much lower than in other treatments and control during the storage period (*p* < 0.05). Abbasi et al. (2023) and Huang et al. (2012) also showed that despite the increase in the number of Enterobacteriaceae during the storage period, coatings containing natural components reduced the growth of Enterobacteriaceae in shrimp.

One of the most important causes of seafood spoilage is psychrophilic bacteria during refrigerated storage (Khaledian et al. 2021). According to Figure 4c, during the storage period, the count of psychrophilic bacteria in all treatments was below the acceptable value (about 7 log CFU/g, Abbasi et al. 2023). At the end of the period, F2 and F3 had significantly fewer psychrophilic bacteria loads compared to F1 and control (*p* < 0.05). Coatings and batter by natural compounds such as fucoidan can act as a barrier to oxygen transport and inhibit bacterial growth. Similar results were reported in the study of Khorami et al. (2024) on rainbow trout fillets coated with chitosan‐alginate/fucoidan. Biswas et al. (2022) also reported similar results on *Pangasius bocourti* fish nuggets coated with dragon fruit peel powder.

The results of mold and yeast growth are shown in Figure 4d. At the beginning of the storage period, the mold and yeast count of treatments was between 3.4 and 4.2 log CFU/g. The amount of mold and yeast in control and F1 increased until day 12, and in F2 and F3 until day 8, and then showed a decreasing trend in all treatments until the end of the storage period. At the end of the storage period, the lowest amount of mold and yeast was observed in F2 and F3, which was significantly different from the control (*p* < 0.05).

Similar to the results of this study, Oppong et al. (2021) reported a decreasing trend in mold and yeast growth in *Pseudotolithus senegalensis* fish nuggets during a 90‐day storage period in the freezer. Biswas et al. (2022) reported a lower count of mold and yeast in *Pangasius bocourti* fish nuggets treated with different amounts of dragon fruit peel powder during 15 days of storage at 5°C.

Polysaccharides such as fucoidan exert their antibacterial effects through two mechanisms. In the first mechanism, the antibacterial effect of fucoidan is mainly attributed to its polyanionic property, in which the sulfate group of the polysaccharide adheres to the bacterial surface, causing cell membrane damage and leakage of nutrients and proteins from the bacterial cell, and ultimately cell death (Wang et al. 2022). The second mechanism is due to the trapping of nutrients by fucoidan. Minerals in food medium with the positively charged are surrounded and trapped by the negatively charged sulfate group of fucoidan, and the bioavailability of nutrients is reduced for bacteria (Ayrapetyan et al. 2021). In addition, structural features such as sugar type, glycosidic bond type, degree of branching, chain dimension, molecular weight, and sulfate content affect the antimicrobial and biological activity of fucoidan (Liu et al. 2017).

### 3.4 Chromaticity Parameters

The final color of seafood products is one of the most important parameters affecting the marketability of these products, which plays an important role in consumer decision‐making (Tomasevic et al. 2019). Therefore, any functional material added to improve the physical and chemical properties of a meat product mustn't cause undesirable changes in its appearance (Banerjee et al. 2020). Table 2 shows the results of the color parameters, L*, a*, and b*, and ΔE, of all treatments during refrigerated storage.

**TABLE 2:** Color changes (*L*, *a* & *b*) of fish nuggets during the refrigerator storage.

	Day					
	Treatment	0	4	8	12	18
L*	Control	26.9 ± 1.5^Ac^	26.7 ± 0.7^Bc^	27.0 ± 1.3^Cc^	35.3 ± 1.3^Aa^	32.6 ± 0.6^Cb^
	F1	25.1 ± 0.6^Bd^	27.4 ± 1.2^Bc^	29.1 ± 1.1^Bb^	35.9 ± 1.4^Aa^	36.6 ± 0.5^Aa^
	F2	26.1 ± 0.6^ABc^	31.9 ± 1.5^Ab^	34.4 ± 0.5^Aa^	34.6 ± 0.6^Aa^	34.8 ± 0.4^Ba^
	F3	25.4 ± 0.9^Bd^	32.6 ± 1.6^Ac^	34.8 ± 0.7^Ab^	32.4 ± 0.5^Bc^	36.4 ± 0.4^Aa^
a*	Control	−2.2 ± 0.8^Cc^	1.8 ± 1.0^Aa^	1.0 ± 0.4^Aa^	0.7 ± 0.7^Aa^	−1.0 ± 1.3^ABb^
	F1	−0.8 ± 0.4^Ac^	0.4 ± 0.6^Bab^	0.9 ± 0.5^Aa^	0.2 ± 0.8^ABab^	−0.2 ± 0.8^Abc^
	F2	−1.7 ± 0.8^BCbc^	−0.6 ± 0.8^Ca^	−0.7 ± 0.4^Ba^	−1.1 ± 0.3^Cab^	−1.9 ± 0.9^Bc^
	F3	−1.0 ± 0.5^ABa^	−1.1 ± 0.8^Ca^	−1.0 ± 0.3^Ba^	−0.5 ± 0.4^BCa^	−0.5 ± 1.0^Aa^
b*	Control	12.6 ± 0.6^Ac^	12.9 ± 0.7^Bc^	13.1 ± 1.0^Cc^	16.6 ± 1.0^Aa^	15.2 ± 1.1^Bb^
	F1	12.3 ± 0.6^Ad^	13.0 ± 1.1^Bcd^	13.7 ± 0.4^Cc^	16.6 ± 1.0^Ab^	17.6 ± 0.8^Aa^
	F2	12.6 ± 0.5^Ab^	13.5 ± 0.9^ABb^	15.2 ± 1.1^Ba^	15.6 ± 0.8^ABa^	13.5 ± 1.5^Cb^
	F3	12.6 ± 0.5^Ad^	14.3 ± 1.2^Ac^	16.6 ± 0.4^Ab^	14.8 ± 0.7^Bc^	18.2 ± 0.5^Aa^
ΔE	Control	1.6 ± 0.4^BCd^	4.1 ± 1.0^BCc^	3.5 ± 0.5^Cc^	9.7 ± 1.2^Aa^	6.6 ± 0.6^Cb^
	F1	2.5 ± 0.4^Ad^	3.0 ± 0.7^Cd^	4.1 ± 0.6^Cc^	10.1 ± 1.4^Ab^	11.1 ± 0.7^Aa^
	F2	1.3 ± 0.6^Cc^	5.4 ± 1.4^ABb^	8.1 ± 0.6^Ba^	8.3 ± 0.6^Ba^	8.1 ± 0.4^Ba^
	F3	2.1 ± 0.6A^Bd^	6.2 ± 1.6^Ac^	8.9 ± 0.6^Ab^	6.2 ± 0.5^Cc^	11.2 ± 0.4^Aa^

*Note:* The effect of the storage time on each treatment is shown in lowercase letters. Comparisons between different treatments are shown in uppercase letters (*p* < 0.05). Data are expressed as mean ± SD. Control (nuggets without fucoidan), F1 (nuggets containing 1% fucoidan), F2 (nuggets containing 2% fucoidan), F3 (nuggets containing 3% fucoidan).

The L* index showed a significant increase (*p* < 0.05) in F3 on days 4, 8, and 18 compared to the control, and also a significant increase in all treatments at the end of the period (*p* < 0.05). Various parameters such as pigments, pH, lipid oxidation, protein denaturation, muscle's physical structure, and microbial spoilage affect the brightness of meaty products (Yu et al. 2018). According to the observed results, the use of fucoidan prevented the discoloration of fish nuggets.

The a* and b* indices are general indices for assessing the freshness of meat products. Some meats, such as pork, have a higher a* index, but in fish, it varies based on species (Yu et al. 2018). In general, the oxidation of the red pigment of hemoglobin and myoglobin and its conversion to brown methemyoglobin are the reasons for the change in meat color, which causes the fish fillet to darken (Li et al. 2017). At the beginning of the period, the a* index was between −2.2 and −0.8, which increased during the period in all treatments except in F2, although these fluctuations were significant only in the control (*p* < 0.05). At the end of the period, F1 and F3 had the highest a* index (*p* > 0.05). The b* index increased during the storage period in all treatments and was not significant only in F2. On days 4, 8, 12, and 18, F3 had a significant increase in b* value compared to the control (*p* < 0.05). Also, the ΔE showed a significant increase in all treatments during the period (*p* < 0.05).

The results of the color parameters obtained in this study were similar to those of da Silva Moreira et al. (2023) on flying fish ( *Hirundichthys affinis* ) burgers treated with different amounts of rosemary, Delfino et al. (2023) on tilapia ( *Oreochromis niloticus* ) burgers treated with 0.2% extracts of Moringa and Lavender, and Rico et al. (2020) on *Trachurus trachurus* burgers treated with chitosan film containing fennel extract. In general, changes in fish nugget color during storage are related to nonenzymatic reactions (microorganisms and oxidation) and enzymes that cause myofibril protein degradation, leading to changes in meat appearance (Cheret et al. 2005).

### 3.5 Sensory Analysis

**FIGURE 5:**
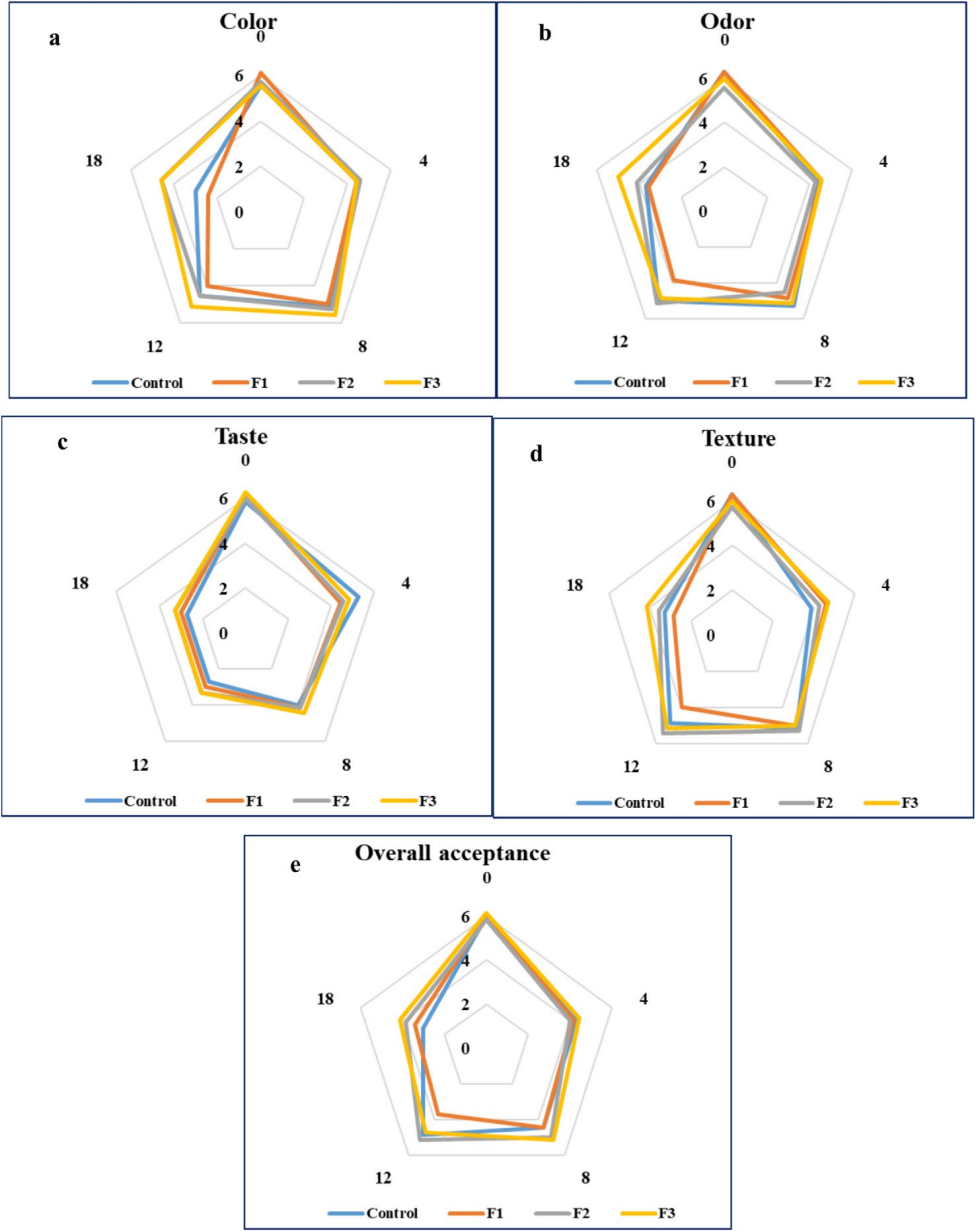
Sensory evaluation of color (a), odor (b), taste (c), texture (d), and overall acceptability (e) of fish nuggets during refrigerator storage. The effect of the storage period on each treatment is shown in lowercase letters. Comparisons between different treatments are shown in uppercase letters (*p* < 0.05). Data are expressed as mean ± SD. Control (nuggets without fucoidan), F1 (nuggets containing 1% fucoidan), F2 (nuggets containing 2% fucoidan), F3 (nuggets containing 3% fucoidan).

Some parameters, such as odor, taste, color, and overall acceptability, are among the most important sensory attributes of food products that affect their acceptance. Figure 5 shows the results of the sensory evaluation of fish nuggets coated with different amounts of fucoidan. The scores of the sensory evaluation decreased in all treatments during the storage period. At the end of the period (day 18), F2 and F3 had the highest scores in color, odor, and texture. In fact, fucoidan incorporated in batter at a higher amount (2% and 3%) performed better on sensory parameters. The taste examination of different nuggets showed that fucoidan did not have a significant effect on the taste of different treatments, and on the final day, F3 was reported to be slightly better. However, in general, after 8 days, the taste in all treatments showed a sharp decline. The overall acceptance results showed that the overall organoleptic properties of the nuggets were preferred on day 0, and F3 received the highest score. Over time, due to the physicochemical changes, the organoleptic properties gradually decreased. On the 18th day of the storage period, control had the lowest overall acceptance score, and F3 had the highest score.

Microbial spoilage and oxidation reactions, which reflect malodorous compounds such as trimethylamines (TMA) and aldehydes, are important factors affecting sensory deterioration (Maqsood and Benjakul 2011). The results of this study showed that F3 had better sensory quality than other treatments, which are related to the antimicrobial and antioxidant properties of fucoidan and its protective effect against oxygen permeation (Dehghan Tanha et al. 2021). In fact, the functional groups of fucoidan (sulfate groups) act as a lipid oxidation inhibitor and as a stabilizing agent. In addition, fucoidan prevented the nuggets from absorbing moisture or dehydrating, which prevented nugget deformation and discoloration (Tosati et al. 2017). The results of the sensory evaluation were in accordance with the chemical and microbial results of this study. Moroney et al. (2013) also showed that the use of brown seaweed extract ( *Laminaria digitata* ) containing laminarin and fucoidan could improve the sensory properties of pork patties. Jannat‐Alipour et al. (2019) reported that the quality and sensory properties of fish surimi formulated with *Ulva intestinalis* algae powder and sulfated polysaccharides were fully maintained during storage, and no negative effects on the organoleptic properties of the formulated products were observed. Dehghan Tanha et al. (2021) showed that the use of an edible coating made of sturgeon skin gelatin and *Portulaca oleracea* extract can effectively improve the sensory properties of sausages made from crucian carp during a 30‐day maintenance at 4°C. They attributed this to the antioxidant and antimicrobial properties of the coating and its protective effect against oxygen penetration.

## 4 Conclusion

In conclusion, the findings of this study show that the addition of fucoidan in fish nugget can effectively increase the shelf life of nuggets during storage at refrigerated temperature (4°C). The fucoidan incorporated in the fish nuggets batter maintained the moisture content of the nuggets and reduced microbial activity during the storage period. The fucoidan‐containing treatments (especially F3) provided a brighter color and maintained better sensory attributes of the nuggets compared to the control group. It can be concluded that incorporating fucoidan as a functional agent in fish nuggets may be a suitable and useful strategy for relatively increasing the shelf life of perishable products such as fish nuggets at refrigerated temperatures.

## Author Contributions

**Mahshid Mojarrad:** investigation, writing – original draft, methodology. **Sedigheh Babaei:** conceptualization, funding acquisition, writing – review and editing, supervision, software, validation, project administration, methodology, investigation, resources. **Tahereh RoshanZamir:** investigation, writing – original draft, methodology. **Mehrnaz Ghanbarzadeh:** writing – original draft, writing – review and editing, methodology, validation, visualization. **Farzaneh Vardizadeh:** investigation, writing – original draft, methodology. **Samaneh Rajabloo:** investigation, writing – original draft, methodology.

## Funding

This Project was funded by the Iran National Elites Foundation [grant number: 771002].

## Conflicts of Interest

The authors declare no conflicts of interest.

## Data Availability

The data that support the findings of this study are available from the corresponding author upon reasonable request.
